# Factors influencing the likelihood of accessing healthcare during the COVID-19 pandemic in Ireland: lessons for the future

**DOI:** 10.12688/hrbopenres.13829.1

**Published:** 2024-02-26

**Authors:** Padraig Buggy, Mary Joyce, Ivan J. Perry, Mary R. Cahill

**Affiliations:** 1 School of Medicine, University College Cork, Cork, County Cork, Ireland; 2 National Suicide Research Foundation, Cork, County Cork, Ireland; 3 School of Public Health, University College Cork, Cork, County Cork, Ireland; 4 Department of Haematology, Cork University Hospital, University College Cork, Cork, Ireland

**Keywords:** COVID-19, Health, Public Health, Hospital, General Practitioner, Healthcare avoidance

## Abstract

**Background:**

Implementation of public health measures during the first wave of the coronavirus disease (COVID-19) pandemic, including travel restrictions and physical distancing, may have impacted population behaviour in seeking medical care. Identifying barriers to accessing healthcare is important, especially for vulnerable groups.

**Methods:**

Data were collected through a nationally representative cross-sectional telephone survey during the first period of easing of COVID-19 restrictions in May and June 2020. Secondary data analysis of the dataset was carried out to examine the factors influencing the likelihood of individuals avoiding General Practitioner (G.P.) and hospital-based care. Poisson regression analyses were conducted to estimate risk ratios with robust variance estimation of the association between selected demographic and self-reported health factors and the risk of avoiding G.P. and hospital-based healthcare.

**Results:**

Of the 969 participants, 152 (15.7%) deliberately avoided contacting their G.P. about non COVID-19 related concerns while 81 (8.4%) reported avoiding hospitals. Three groups, women (Rate Ratio (RR): 1.75, 95% Confidence Interval (CI): 1.28 – 2.40), individuals who reported experiencing an adverse life event within the last 3 months (RR: 1.79, 95% CI: 1.33 – 2.40), and those at an increased risk of infection (RR: 1.65, 95% CI: 1.06 – 2.58) were more likely to avoid contact with their G.P. Individuals at a higher risk of avoiding hospital-based care were those aged 50 – 59 years (RR: 2.27, 95% CI: 1.03 – 4.98) and 70 years+ (RR: 2.73, 95% CI: 1.24 – 6.01), individuals at an increased risk of infection (RR: 2.07, 95% CI: 1.20 – 3.56), smokers (RR: 1.68, 95% CI: 1.02 – 2.77) and those who agreed they were “likely to catch COVID-19” (RR: 2.80, 95% CI: 1.25 – 6.25).

**Conclusion:**

These findings highlight the importance of public health awareness and education regarding accessing healthcare during a pandemic and should be considered in future pandemic preparedness.

## Introduction

The World Health Organization (WHO) declared coronavirus disease (COVID-19) a global pandemic in March 2020. Between March 1 ^st^ 2020, and January 7 ^th^ 2023, Ireland registered 1,697,775 COVID-19 cases and 8,309 deaths since the start of the pandemic ^1^. In line with WHO guidelines to suppress the spread of the virus, public health measures, including instructions to stay at home, travel restrictions, and physical distancing, were implemented in Ireland ^2^. We asked whether these measures, introduced to protect the public, may have impacted non COVID-19 related medical care and population well-being.

A decrease in the number of attendances to Irish public hospitals providing emergency care was reported in March 2020 compared to January and February of the same year and compared with figures from March 2019 ^3^, substantiating the hypothesis that the pandemic and associated restrictions had an impact on the health behaviours of the population. This trend has also been reported in previous studies. A study of one million medical admissions in the U.S. revealed a decline in non COVID-19 related admissions of 20% at the beginning of the pandemic between February and April 2020, including reductions in presentations of serious life-threatening conditions such as sepsis (25% reduction in the 3 month period), acute ST-elevation myocardial infarction (22%), and pneumonia (40%) ^4^. Similar results were observed in another US study, where during the pandemic, a 23% reduction was observed in ED presentations of myocardial infarction, 20% for strokes, and a 10% reduction in hyperglycaemic crises. The authors suggested that the most plausible explanation was that patients could not access care or avoided or delayed seeking care ^5^. A study in the UK revealed that 44.8% of the population reported having potential malignancy-related symptoms during the pandemic (n=3025) and did not seek medical attention ^6^. These findings suggest that individuals’ health-seeking behaviours changed during the pandemic, even in the context of significant conditions that would normally result in an emergency presentation to the hospital.

One US-based study demonstrated that higher risk perception was a strong predictor of medical avoidance ^7^. Risk perception was measured using participants’ responses to three questions in an online survey completed by Massachusetts Institute of Technology (MIT) researchers: risk of COVID-19 to their community, perceived risk of infection, and infection severity. Significant predictors of higher risk perceptions included female sex, knowing someone with COVID-19, older age, and poorer health. A higher risk perception was associated with avoiding medical care ^7^.

In the aftermath of the pandemic, the consequences of health care avoidance became evident. A study in the US compared the first presentations of early- and late-stage breast and colorectal cancer before and during the COVID pandemic (comparing 2019 figures to 2020). That study reported a statistically significant decrease in the number of women presenting with stage 1 breast cancer but a significant increase in those presenting with stage 4 breast cancer during the pandemic. A continuation of this trend during the early months of 2021 was also observed ^8^. A further study in the UK also demonstrated that patients presented with higher stages of breast cancer and more node-positive and metastatic disease on initial presentation and diagnosis in 2020 than in the pre-pandemic period in 2019 ^9^.

This study aimed to examine the influence of demographic and self-reported health factors on health-seeking behaviours, specifically the likelihood of avoiding general practice (G.P.) and hospital-based healthcare, in the Irish population during the initial stages of the COVID-19 pandemic.

## Methods

### Study design

This study was part of a larger study aimed at estimating the effects of public health measures in the Republic of Ireland during the COVID-19 pandemic ^10^. A nationally representative cross-sectional telephone survey was conducted to assess knowledge, attitudes, and compliance with physical distancing measures as well as physical, mental, and social well-being. The survey was conducted between May 26 ^th^ – June 17 ^th^, 2020, during the initial easing of restrictions in Ireland. The marketing company IPSOS MRBI conducted a telephone survey on behalf of the School of Public Health at UCC. This study used secondary data drawn from the cross-sectional telephone survey described above.

### Participants

Participants in the survey were sampled from the general population. The inclusion criteria were as follows: aged ≥ 18 years, residing in Ireland, and having a telephone (landline or mobile telephone number). To achieve a nationally representative sample, surveys were conducted using random digit-dialling (approximately 80% mobile, 20% landline) with an estimated response rate of 43.6%, accounting for non-operational and non-answering numbers. An a priori sample size calculation of 1,000 participants, excluding non-responders, non-operational numbers, and non-answering numbers, produced a two-sided 95% Confidence Interval (CI) with a width of 0.028 when the sample proportion was 0.05.

### Measures

The survey gathered information related to participants’ physical health, mental health, and social well-being as well as their socio-demographic characteristics ^11^. A full list of the primary data items can be found in Troya *et al.* 2020 ^10^.

The following sociodemographic variables were included in this secondary data analysis: gender, age group, education level, employment status, and income level. Health-related variables extracted were general health status, alcohol and tobacco consumption, recent stressful life events, participants’ perceptions of COVID-19 (as a serious illness), and perception of the likelihood of contracting the virus. Information about whether participants cocooned during the restrictions and reasons for cocooning were also extracted. Finally, healthcare-seeking behaviours specifically related to G.P. and hospital care were examined.

A new variable was created to identify individuals who were at an increased risk of infection. This variable was created based on responses to the questions asked about cocooning. Individuals were categorized as having an increased risk of infection if they cocooned because of diabetes, cancer, a severe respiratory condition, a condition with a very high risk of infections, or being on medication that increased the likelihood of contracting infections.

### Statistical analyses

The statistical software packages IBM SPSS Version 27 and Stata Version 15.1 were used to analyse the data. Descriptive statistics summarized the selected sociodemographic characteristics as well as proportions of those who (a) avoided contact with G.P. and (b) avoided hospital-based healthcare.

Poisson regression analyses were conducted to estimate the risk ratios and 95% CIs with robust variance estimation of the association between selected demographic factors, health- and lifestyle-related factors, and the risk of avoiding G.P. or hospital-based healthcare. Survey commands were used and the estimates were weighted to account for the survey sampling design. The significance level was set at *p* < 0.05.

### Ethics

Ethical approval for the study was obtained from the Clinical Research Ethics Committee of the Cork Teaching Hospitals (Ref: EMC4 (b)05/05/20) in April 2020. Informed verbal consent was obtained by the interviewer before proceeding with the survey. Further information regarding ethical considerations and informed consent can be found in Troya *et al.* ^8^. Ethics approval for the secondary data analysis was obtained from the Clinical Research Ethics Committee of the Cork Teaching Hospitals in November 2021 (Ref: & ECM 3 (fff) 16/11/2021).

## Results

Data from 969 participants were analysed ^12^. The sociodemographic characteristics of the participants are provided in Table 1.

**Table 1.** Socio-demographic characteristics of participants.

	Frequency (%)
**Gender**	
Men	466 (48.1)
Women	501 (51.7)
Other	2 (0.2)
**Age group**	
18–29 years	167 (17.2)
30–39 years	167 (17.2)
40–49 years	182 (18.8)
50–59 years	157 (16.2)
60–69 years	157 (16.2)
70 years +	127 (13.1)
Missing	12 (1.2)
**Highest level of education**	
Primary Level	41 (4.2)
Group/ Inter/ Junior Certificate	76 (7.8)
Leaving Certificate	188 (19.4)
Other Second Level/ PLC Cert or similar	92 (9.5)
Third Level Degree/ Postgraduate Course	566 (58.4)
Other/ don't know	6 (0.6)
**Employment status**	
Working as employee (full-time or part-time)	500 (51.6)
Self-employed	90 (9.3)
Unemployed/ Seeking work	81 (8.4)
Not working due to permanent illness/ disability	41 (4.2)
Retired	186 (19.2)
Full-time homemaker/ looking after family	29 (3.0)
Student	34 (3.5)
Other/ don't know	8 (0.8)
**Annual combined net income for household**	
Under €19,999	115 (11.9)
€20,000 to €29,999	109 (11.2)
€30,000 to €49,999	227 (23.4)
€50,000 to €79,999	206 (21.3)
€80,000 or greater	128 (13.2)
Don’t know/ Refused to answer	184 (19.0)

There were similar numbers of men and women (51.7%). Participants ranged in age from 18–91 years, with a mean age of 47.9 (SD = 17.2). Over half of the sample had completed third-level education and worked as employees (either full-time or part-time).

### Healthcare behaviour during COVID-19 restrictions

Participants were asked about their healthcare behaviours during the first period of the restricted movement/ lockdown. Of the 969 participants, 152 (15.7%) deliberately avoided contact with their G.P. about non COVID-19 related concerns while 81 (8.4%) avoided going to the hospital with a non COVID-19 related concern or health problem. The sociodemographic characteristics of the participants who avoided G.P. and hospital-based healthcare are outlined in Table 2.

**Table 2.** Socio-demographic characteristics of participants who avoided G.P. and hospital-based healthcare.

	Avoided G.P. (n=152) N (%)	Avoided hospital-based healthcare (n=81) N (%)
**Gender**		
Men ( *n* = 466)	51 (10.9)	28 (6.0)
Women ( *n* = 501)	100 (20.0)	53 (10.6)
**Age group**		
18–29 years ( *n* = 167)	23 (13.8)	7 (4.2)
30–39 years ( *n* = 167)	29 (17.4)	14 (8.4)
40–49 years ( *n* = 182)	37 (20.3)	16 (8.8)
50–59 years ( *n* = 157)	28 (17.8)	19 (12.1)
60–69 years ( *n* = 157)	20 (12.7)	7 (4.5)
70 years + ( *n* = 127)	13 (10.2)	17 (13.4)
**Highest level of education**		
Primary Level & Group/ Inter/ Junior Certificate ( *n* = 117)	12 (10.3)	16 (13.7)
Leaving Certificate ( *n* = 188)	27 (14.4)	19 (10.1)
Other Second Level/ PLC Cert or similar ( *n* = 92)	11 (12.0)	8 (8.7)
Third Level Degree/ Postgraduate Course ( *n* = 566)	101 (17.8)	38 (6.7)
**Employment status**		
Working as employee (full-time or part-time) ( *n* = 500)	79 (15.8)	37 (7.4)
Self-employed ( *n* = 90)	12 (13.3)	10 (11.1)
Unemployed/ Seeking work ( *n* = 81)	18 (22.2)	3 (3.7)
Not working due to permanent illness/ disability ( *n* = 41)	11 (26.8)	5 (12.2)
Retired ( *n* = 186)	21 (11.3)	17 (9.1)
Full-time homemaker/ looking after family ( *n* = 29)	6 (20.7)	5 (17.2)
Student ( *n* = 34)	4 (11.8)	4 (11.8)

One-fifth of all female participants ( *n* = 100; 20.0%) and those aged 40–49 years ( *n* = 37; 20.3%) reported avoiding contact with their G.P. More than a quarter of the participants who were not working due to illness/ disability ( *n* = 11; 26.8%) also avoided contact with their G.P., although the overall number of participants in this grouping was small.

Just over 10% of all female participants ( *n* = 53) and 13% of those aged 70 years and above ( *n* = 17) reported avoiding going to hospital for non COVID-19 related concerns.

Information on health and lifestyle factors, as well as COVID-19 related perceptions was also examined. The self-reported health factors and COVID-19 perceptions of participants who avoided G.P. and hospital-based healthcare are outlined in Table 3.

**Table 3.** Health and lifestyle factors and COVID-19 related perceptions of participants who avoided G.P. and hospital-based healthcare.

	Avoided G.P. (n=152) N (%)	Avoided hospital-based healthcare (n=81) N (%)
**General health status**		
Excellent ( *n* = 163)	17 (10.4)	7 (4.3)
Very good ( *n* = 335)	48 (14.3)	18 (5.4)
Good ( *n* = 321)	48 (15.0)	28 (8.7)
Fair ( *n* = 118)	27 (22.9)	19 (16.1)
Poor ( *n* = 31)	12 (38.7)	9 (29.0)
**Alcohol consumption**		
Do not drink alcohol ( *n* = 227)	40 (17.6)	19 (8.4)
Occasional drinker ( *n* = 471)	63 (13.4)	43 (9.1)
Moderate drinker ( *n* = 251)	45 (17.9)	17 (6.8)
Heavy drinker ( *n* = 20)	4 (20.0)	2 (10.0)
**Smoking**		
Yes ( *n* = 151)	28 (18.5)	18 (11.9)
No ( *n* = 818)	124 (15.2)	63 (7.7)
**Adverse life event (<3 months)**		
Yes ( *n* = 287)	70 (24.4)	32 (11.1)
No ( *n* = 682)	82 (12.0)	49 (7.2)
**Increased risk of infection**		
Yes ( *n* = 56)	18 (32.1)	14 (25.0)
No ( *n* = 910)	134 (14.7)	67 (7.3)
**Perceived likelihood of contracting coronavirus disease (COVID-19)**		
Strongly agree ( *n* = 123)	21 (17.1)	12 (9.8)
Tend to agree ( *n* = 292)	55 (18.8)	32 (11.0)
Tend to disagree ( *n* = 365)	52 (14.2)	27 (7.4)
Strongly disagree ( *n* = 165)	20 (12.1)	9 (5.5)
Don’t know ( *n* = 24)	4 (16.7)	1 (4.2)
**Perceived likelihood that COVID-19 would be serious illness**		
Strongly agree ( *n* = 406)	69 (17.0)	48 (11.8)
Tend to agree ( *n* = 283)	52 (18.4)	19 (6.7)
Tend to disagree ( *n* = 199)	19 (9.5)	11 (5.5)
Strongly disagree ( *n* = 68)	10 (14.7)	3 (4.4)
Don’t know ( *n* = 13)	2 (15.4)	0 (0)

More than a third of individuals who rated their health as ‘poor’ ( *n* = 12; 38.7%) and almost a third of those who were at an increased risk of infection ( *n* = 18, 32.1%) avoided G.P. contact. Similar observations were noted for other healthcare behaviours with 29% of those who reported ‘poor’ health and a quarter of those at an increased risk of infection avoiding hospital-based healthcare.

Findings from the Poisson regression analysis indicated that there was a significantly higher risk of avoiding contact with G.P. for women than for men (Rate Ratio (RR): 1.75 (1.28 – 2.40) (see Table 4). There was a significantly higher risk of avoiding G.P. contact for those who reported experiencing an adverse life event in the previous three months (RR: 1.79 (1.33 – 2.40), and for those at an increased risk of infection RR: 1.65 (1.06 – 2.58).

**Table 4.** Risk ratios (RR) for avoiding GP based healthcare.

	Crude		Adjusted	
Variable	Risk ratio (confidence interval)	P value	Risk ratio (confidence interval)	P value
**Gender**				
Men	Reference			
Women	2.03 (1.41 – 2.93)	<0.001	1.75 (1.28 - 2.40)	<0.001
**Age group**				
18–29	Reference			
30–39	1.32 (0.73 – 2.39)	0.366	1.32 (0.81 - 2.14)	0.263
40–49	1.62 (0.92 – 2.86)	0.097	1.45 (0.90 – 2.33)	0.127
50–59	1.37 (0.75 – 2.50)	0.305	1.16 (0.70 – 1.93)	0.572
60–69	0.91 (0.48 – 1.74)	0.784	0.92 (0.52 - 1.62)	0.772
70–100	0.71 (0.35 – 1.47)	0.361	0.69 (0.36 - 1.32)	0.261
**Health status** *				
Excellent	Reference		N/A	
Very good	1.45 (0.80 - 2.60)	0.219	N/A	N/A
Good	1.52 (0.84 - 2.73)	0.166	N/A	N/A
Fair	2.55 (1.32 - 4.93)	0.006	N/A	N/A
Poor	5.42 (2.25 - 13.08)	<0.001	N/A	N/A
**Alcohol consumption**				
Do not drink	Reference			
Occasional drinker	0.72 (0.46 – 1.10)	0.130	0.66 (0.46 – 0.95)	0.026
Moderate drinker	1.01 (0.63 – 1.62)	0.966	1.07 (0.73 – 1.57)	0.725
Heavy drinker	1.16 (0.37 – 3.64)	0.804	0.94 (0.35 – 2.57)	0.908
**Smoking**				
No	Reference			
Yes	1.28 (0.81 – 2.01)	0.284	1.12 (0.78 - 1.61)	0.545
**Adverse life event (<3 months)**				
No	Reference			
Yes	2.38 (1.67 – 3.39)	<0.001	1.79 (1.33 – 2.40)	<0.000
**Increased risk of infection**				
No	Reference			
Yes	2.74 (1.52 – 4.95)	0.001	1.65 (1.06 - 2.58)	0.027
**COVID would be a serious illness for me**				
Strongly disagree	Reference			
Tend to disagree	0.61 (0.27 – 1.39)	0.241	0.47 (0.23 – 0.96)	0.039
Tend to agree	1.32 (0.63 – 2.75)	0.463	0.97 (0.51 – 1.86)	0.926
Strongly agree	1.19 (0.58 - 2.45)	0.634	1.00 (0.52 – 1.93)	0.997
**I am likely to catch COVID**				
Strongly disagree	Reference			
Tend to disagree	1.20 (0.69 – 2.09)	0.509	1.14 (0.69 – 1.88)	0.609
Tend to agree	1.69 (0.97 – 2.93)	0.063	1.53 (0.94 – 2.50)	0.086
Strongly agree	1.52 (0.78 – 2.96)	0.214	1.19 (0.67 – 2.14)	0.553

* Self-reported health status was not considered in the multivariate analysis due to collinearity with increased risk of infection.

In relation to hospital-based care, there was a significantly higher risk for individuals aged 50 – 59 years, RR: 2.27 (1.03 – 4.98), and those aged 70 years and above, RR: 2.73 (1.24 – 6.01) of avoiding going to hospital than those aged 18–29 years (see Table 5). There was also a significantly higher risk of avoiding going to hospital for those who were at an increased risk of infection (RR: 2.07 (1.20 – 3.56) and those who were smokers (RR: 1.68 (1.02 – 2.77). Lastly, participants who agreed that they were likely to catch COVID-19 were at a higher risk of avoiding going to hospital than those who strongly disagreed (RR: 2.80 (1.25 – 6.25).

**Table 5.** Risk ratios (RR) for avoiding hospital-based healthcare.

	Crude		Adjusted	
Variable	Risk ratio (confidence interval)	P value	Risk ratio (confidence interval)	P value
**Gender**				
Men	Reference			
Women	1.85 (1.15 - 2.99)	0.011	1.49 (0.93 - 2.37)	0.094
**Age**				
18–29	Reference			
30–39	2.11 (0.83 – 5.36)	0.118	1.92 (0.84 – 4.40)	0.123
40–49	2.20 (0.88 – 5.50)	0.090	1.90 (0.84 – 4.30)	0.124
50–59	3.15 (1.28 – 7.71)	0.012	2.27 (1.03 – 4.98)	0.041
60–69	1.07 (0.37 – 3.11)	0.906	0.96 (0.36 – 2.60)	0.939
70–100	3.53 (1.42 – 8.80)	0.007	2.73 (1.24 – 6.01)	0.013
Health status *				
Excellent	Reference		N/A	
Very good	1.27 (0.52 - 3.10)	0.601	N/A	N/A
Good	2.13 (0.91 - 4.99)	0.082	N/A	N/A
Fair	4.28 (1.73 - 10.55)	0.002	N/A	N/A
Poor	9.12 (3.08 - 26.95)	<0.000	N/A	N/A
**Alcohol consumption**				
Do not drink	Reference			
Occasional drinker	1.10 (0.63 – 1.93)	0.741	1.16 (0.69 – 1.94)	0.584
Moderate drinker	0.80 (0.40 – 1.58)	0.517	0.91 (0.48 – 1.71)	0.764
Heavy drinker	1.22 (0.26 – 5.64)	0.802	1.35 (0.35 – 5.20)	0.660
**Smoking**				
No	Reference			
Yes	1.62 (0.93 – 2.82)	0.089	1.68 (1.02 – 2.77)	0.043
**Adverse life event (<3 months)**				
No	Reference			
Yes	1.62 (1.01 – 2.59)	0.044	1.43 (0.93 – 2.18)	0.102
**Increased risk of infection**				
No	Reference			
Yes	4.20 (2.19 – 8.09)	<0.001	2.07 (1.20 – 3.56)	0.009
**COVID would be a serious illness for me**				
Strongly disagree	Reference			
Tend to disagree	1.27 (0.34 – 4.69)	0.722	0.74 (0.21 – 2.65)	0.648
Tend to agree	1.57 (0.45 – 5.45)	0.481	0.84 (0.25 – 2.77)	0.771
Strongly agree	2.91 (0.88 – 9.61)	0.081	1.42 (0.44 – 4.56)	0.552
**I am likely to catch COVID**				
Strongly disagree	Reference			
Tend to disagree	1.39 (0.64 – 3.02)	0.408	1.92 (0.88 – 4.19)	0.099
Tend to agree	2.13 (0.99 – 4.59)	0.052	2.80 (1.25 – 6.25)	0.012
Strongly agree	1.87 (0.76 – 4.60)	0.170	1.91 (0.80 – 4.57)	0.143

* Self-reported health status was not considered in the multivariate analysis due to collinearity with increased risk of infection.

## Discussion

Our study of the factors influencing the likelihood of accessing healthcare during the initial months of the COVID pandemic in Ireland has identified several significant findings with actionable lessons for the future. This study identified subgroups of individuals who were at a greater risk of healthcare avoidant behaviours during the initial period of easing COVID-19 restrictions in Ireland. Women, and those identified as being at an increased risk of infection, as well as individuals who reported experiencing an adverse life event in the previous three months, were at a higher risk of avoiding contact with their G.P. about non COVID-19 related concerns. Individuals at a higher risk of avoiding hospital-based care were those aged 50–59 years, adults over 70 years, individuals at an increased risk of infection, smokers, and those who agreed that they were likely to become infected with COVID-19.

Our findings replicate those of a US study, in which women and older adults were also more likely to avoid seeking healthcare. The study also showed that a higher perceived risk of infection is associated with a higher probability of avoiding medical care ^7^. There is evidence from a UK study of failure to seek medical attention during the pandemic among individuals with potential malignancy-related symptoms ^13^.

A Dutch study also identified that women and individuals with lower perceived overall health and mental health issues (depression and anxiety) avoided seeking hospital-based healthcare, even when these groups had potentially serious symptoms (limb weakness, palpitations, and chest pain) ^14^. Taken as a whole, including our findings in the Irish population, studies clearly demonstrate an avoidance in healthcare-seeking behaviour from the beginning of the pandemic in several vulnerable groups.

For individuals to feel safe and able to access healthcare in future pandemics in Ireland, it is crucial that we note and learn the lessons from the COVID emergency, here in this country and elsewhere. The shift in the behaviour of some vulnerable groups in our population during the pandemic suggests that individuals appear to be weighing the risk of attending hospitals or GP surgery as higher than the risk of remaining at home with potentially serious issues. Post-pandemic studies have shown that these issues include late-stage, potentially curable, cancers ^8^. In Ireland, in 2020, the National Cancer Registry reported a 10% shortfall in expected new diagnoses of cancer, improving to a 6% shortfall in 2021 ^15^.

Our data provide important information specific to Ireland on behaviours during the pandemic. Because this study was carried out during periods of lockdown, recall bias was minimized and respondents answered regarding behaviours in real time. These valuable data provide the basis for targeted campaigns, specific information, and encouragement for vulnerable groups to help improve outcomes in future pandemics. The findings from this study suggest that people who may be most in need of health care are those who are least likely to access it.

These findings are especially relevant to patients with blood cancer, where the nature of the cancer, which affects the marrow and immune system, means that they are the most vulnerable group to opportunistic infections. Anecdotally, immunocompromised haematology patients frequently required telephone discussion and reassurance prior to hospital attendance during the pandemic and management of a structurally separate non -COVID pathway within the hospital setting while receiving emergency care for infection/ possible sepsis during the pandemic. 

This study had several limitations. The estimated response rate was relatively high for a population-based telephone survey (43.6%). However, individuals who did not respond to the survey, for whatever reason, may have responded differently to participants who responded to the survey, creating a response bias. Our study population may underrepresent the elderly population or the homeless community, who may be less likely to have access to a phone or respond to a telephone survey.

Responders’ reports of being at risk of infection are based on self-perception and may or may not be based on their medical diagnoses. However, this has the advantage of representing the respondents’ understanding of their own situation, which is a likely determinant of decisions, regardless of any objective underlying diagnoses.

Lessons can be drawn for future pandemic preparedness in Ireland. Our data supports the use of advance planning to target vulnerable groups with appropriate information and reassurance regarding safety measures for care. Planning could include infrastructure for pivoting quickly to more telephone clinics, preparing for video consultations, and laying the necessary groundwork for any data sharing issues, including utilizing artificial intelligence where appropriate. Addressing the technical requirements in advance would be helpful. All of these measures could assist in managing emergent pathogens in the groups shown to avoid hospital and healthcare settings. Planning for effective communication between these groups and general practitioners is also prudent. In Ireland, the coexistence of the cyber attack on the HSE during the pandemic also hampered efforts to provide appropriate on-site or remote care. Contingency planning for multiple simultaneous threats is required in the current global climate.

## Data Availability

Zenodo: Factors influencing the likelihood of accessing healthcare during the COVID-19 pandemic in Ireland: lessons for the future. https://doi.org/10.5281/zenodo.10073077 ^12^ This project contains the following underlying data: National Household Survey – Wave 1, Ver. 2.sav Harvard Dataverse: Questionnaires for Surveys WP1 and WP2. https://doi.org/10.7910/DVN/EKUTFF ^11^ This project contains the following extended data: Survey 1 questionnaire in DOCX format (Appendix I) Survey 2 questionnaire in DOCX format (Appendix II) Data are available under the terms of the Creative Commons Attribution 4.0 International license (CC-BY 4.0).
