# Factors associated with self-reported healthcare utilization avoidance during the COVID-19 pandemic in Ireland: lessons for the future

**DOI:** 10.12688/hrbopenres.13829.2

**Published:** 2025-10-24

**Authors:** Padraig Buggy, Mary Joyce, Ivan J. Perry, Mary R. Cahill

**Affiliations:** 1 School of Medicine, University College Cork, Cork, County Cork, Ireland; 2 National Suicide Research Foundation, Cork, County Cork, Ireland; 3 School of Public Health, University College Cork, Cork, County Cork, Ireland; 4 Department of Haematology, Cork University Hospital, University College Cork, Cork, Ireland

**Keywords:** COVID-19, Health, Public Health, Hospital, General Practitioner, Healthcare avoidance

## Abstract

**Background:**

Implementation of public health measures during the first wave of the coronavirus disease (COVID-19) pandemic, including travel restrictions and physical distancing, may have impacted population behaviour in seeking medical care. Identifying factors associated with healthcare avoidance is important, especially for vulnerable groups.

**Methods:**

A nationally representative cross-sectional telephone survey addressing the impact of public health restrictions on physical, mental and social wellbeing, was conducted during the first period of easing of COVID-19 restrictions in May and June 2020. Secondary data analysis of the dataset was carried out to examine the factors associated with self-reported deliberate/conscious avoidance of General Practitioner (G.P.) and hospital-based care. Poisson regression analyses were conducted to estimate risk ratios with robust variance estimation of the association between selected demographic and self-reported health factors and the risk of avoiding G.P. and hospital-based healthcare.

**Results:**

Of the 969 participants, 152 (15.7%) deliberately avoided contacting their G.P. about non COVID-19 related concerns while 81 (8.4%) reported avoiding hospitals. In multivariate analyses, three groups, women (Rate Ratio(RR): 1.77, 95% Confidence Interval (CI): 1.30 – 2.43), individuals who reported experiencing an adverse life event within the previous 3 months (RR: 1.70, 95% CI: 1.27 – 2.28), and those with self-reported poor health status (trend *p* < 0.001) were more likely to report that they avoided contact with their G.P. Individuals at a higher risk of avoiding hospital-based care were older (trend with age, *p =* 0.063), those who tended to agree they were “likely to catch COVID-19” (trend *p* = 0.052), and those with self-reported poor health status (trend *p* <0.001).

**Conclusion:**

These findings highlight the importance of public health awareness and education regarding accessing healthcare during a pandemic and should be considered in future pandemic preparedness.

## Introduction

The World Health Organization (WHO) declared coronavirus disease (COVID-19) a global pandemic in March 2020. Between March 1 ^st^ 2020, and January 7 ^th^ 2023, Ireland registered 1,697,775 COVID-19 cases and 8,309 deaths from COVID-19 ^1^. Ireland implemented a range of public health measures to suppress the spread of the virus, including instructions to stay at home, travel restrictions, and physical distancing, consistent with WHO and other international guidelines ^2^. The focus of this research was on the impact of these measures on self-reported health seeking behaviour in the population. While it was anticipated that these measures would reduce presentations with non COVID-19 related illness in both primary care and hospital settings, the magnitude and distribution of these effects is not well described.

A decrease in the number of attendances to Irish public hospitals providing emergency care was reported in March 2020 compared to January and February of the same year and compared with figures from March 2019 ^3^. This trend has also been reported in other countries. A study of one million medical admissions in the U.S. revealed a decline in non COVID-19 related admissions of 20% at the beginning of the pandemic between February and April 2020, including reductions in presentations of serious life-threatening conditions such as sepsis (25% reduction in the 3 month period), acute ST-elevation myocardial infarction (22%), and pneumonia (40%) ^4^. A similar trend was observed in another US study, where during the pandemic, a 23% reduction was observed in ED presentations of myocardial infarction, 20% for strokes, and a 10% reduction in hyperglycaemic crises. The authors suggested that the most plausible explanation was that patients could not access care or avoided or delayed seeking care ^5^. A study in the UK revealed that 45% of the population reported having potential malignancy-related symptoms during the pandemic (n=3025) and did not seek medical attention ^6^. These findings suggest that individuals’ health-seeking behaviours changed during the pandemic, even in the context of significant conditions that would normally result in an emergency presentation to the hospital.

In a further US-based study, higher COVID-19 risk perception was a strong predictor of medical avoidance ^7^. Risk perception was assessed using participants’ responses to three questions in an online survey: risk of COVID-19 to their community, perceived risk of infection, and perceived severity if the respondent became infected. Significant predictors of higher risk perceptions included female sex, knowing someone with COVID-19, older age, and poorer health.

In the aftermath of the pandemic, the consequences of health care avoidance became evident. A study in the US compared the first presentations of early- and late-stage breast and colorectal cancer before and during the COVID pandemic (comparing 2019 figures to 2020). That study reported a statistically significant decrease in the number of women presenting with stage 1 breast cancer but a significant increase in those presenting with stage 4 breast cancer during the pandemic. A continuation of this trend during the early months of 2021 was also observed ^8^. A further study in the UK also demonstrated that patients presented with higher stages of breast cancer and more node-positive and metastatic disease on initial presentation and diagnosis in 2020 than in the pre-pandemic period in 2019 ^9^.

This study aimed to examine the influence of socio-demographic factors, self-reported health status, health related behaviours and exposures, and COVID-19 risk perception on self-reported health-seeking behaviours, specifically the likelihood of avoiding primary care, i.e. general practice (G.P.) and hospital-based healthcare, in the Irish population during the initial stages of the COVID-19 pandemic.

## Methods

### Study design

This study is based on secondary analyses of a larger study aimed at estimating the effects of public health measures in the Republic of Ireland during the COVID-19 pandemic ^10^. A nationally representative cross-sectional telephone survey was conducted to assess knowledge, attitudes, and compliance with physical distancing measures as well as physical, mental, and social well-being. The survey was conducted between May 26 ^th^ – June 17 ^th^, 2020, during the initial easing of restrictions in Ireland. The marketing company IPSOS MRBI conducted a telephone survey on behalf of the School of Public Health at UCC.

### Participants

Participants in the survey were sampled from the general population. The inclusion criteria were as follows: aged ≥ 18 years, residing in Ireland, and having a telephone (landline or mobile telephone number). To achieve a nationally representative sample, surveys were conducted using random digit-dialling (approximately 80% mobile, 20% landline) with an estimated response rate of 43.6% based on non-operational and non-answering numbers ^11^. Data were weighted by age, gender, and region, with population estimates based on the Irish Labour Force Survey ^12^.

### Measures

The survey gathered information related to participants’ physical health, mental health, and social well-being as well as their socio-demographic characteristics ^10^. A full list of the primary data items can be found in Troya *et al.* 2020 ^13^.

The following sociodemographic variables were included in this secondary data analysis: gender, age group, education level, employment status, and income level. Health-related variables extracted included general health status, alcohol and tobacco consumption, recent stressful life events, participants’ perceptions of COVID-19 (as a serious illness), and perception of the likelihood of contracting the virus. During the pandemic, the Irish Government asked persons aged over 70 years and those highly vulnerable to COVID-19 infection for other reasons such as chronic disease to “cocoon”, i.e. remain confined to home with minimal face-to-face contact with those from outside their home. Data on whether participants cocooned during the restrictions and reasons for cocooning were also extracted from the dataset. A new variable was created to identify individuals who were at an increased risk of infection. This variable was created based on responses to the questions asked about cocooning. Individuals were categorized as having an increased risk of infection if they cocooned because of diabetes, cancer, a severe respiratory condition, a condition with a very high risk of infections, or being on medication that increased the likelihood of contracting infections. The outcome variables in these analyses were self-reported healthcare-avoidant behaviours specifically related to G.P. and hospital care based on the following questions: *During the period of restricted movement/lockdown, have you done any of the following: “Deliberately/consciously avoided contacting your GP (General Practitioner) about non-coronavirus concerns or problems that you would normally bring to his/her attention.”* and *“Deliberately/consciously avoided going to the hospital with a non-coronavirus concern or health problem that would in normal circumstances require a visit to the hospital*.”

### Statistical analyses

The statistical software packages IBM SPSS Version 27 and Stata Version 15.1 were used to analyse the data. Descriptive statistics summarized the selected sociodemographic characteristics as well as proportions of those who (a) avoided contact with G.P. and (b) avoided hospital-based healthcare.

Poisson regression analyses were conducted to estimate the risk ratios and 95% CIs with robust variance estimation of the association between selected demographic factors, health- and lifestyle-related factors, and the risk of avoiding G.P. or hospital-based healthcare in both univariate and multivariate analyses. Survey commands were used and the estimates were weighted to account for the survey sampling design. The significance level was set at *p* < 0.05. In the multivariate analyses presented in Table 4 and Table 5, each variable was adjusted for all of the other factors presented in these tables.

There were a small number (<5) of ‘Don’t know’ responses for the following variables which were coded as missing and removed for the analyses: general health status, healthcare avoidance behaviours related to G.P. and hospital care.

### Ethics

Ethical approval for the study was obtained from the Clinical Research Ethics Committee of the Cork Teaching Hospitals (Ref: EMC4 (b)05/05/20) in April 2020. Informed verbal consent was obtained by the interviewer before proceeding with the survey. Further information regarding ethical considerations and informed consent can be found in Troya *et al.* ^10^. Ethics approval for the secondary data analysis was obtained from the Clinical Research Ethics Committee of the Cork Teaching Hospitals in November 2021 (Ref: & ECM 3 (fff) 16/11/2021).

## Results

Data from 969 participants were analysed. The sociodemographic characteristics of the participants are provided in Table 1.

**Table 1.** Socio-demographic characteristics of participants.

	Frequency (%)
**Gender**	
Men	466 (48.1)
Women	501 (51.7)
Other	2 (0.2)
**Age group**	
18–29 years	167 (17.2)
30–39 years	167 (17.2)
40–49 years	182 (18.8)
50–59 years	157 (16.2)
60–69 years	157 (16.2)
70 years +	127 (13.1)
Missing	12 (1.2)
**Highest level of education**	
Primary Level	41 (4.2)
Group/ Inter/ Junior Certificate	76 (7.8)
Leaving Certificate	188 (19.4)
Other Second Level/ PLC Cert or similar	92 (9.5)
Third Level Degree/ Postgraduate Course	566 (58.4)
Other/ don't know	6 (0.6)
**Employment status**	
Working as employee (full-time or part-time)	500 (51.6)
Self-employed	90 (9.3)
Unemployed/ Seeking work	81 (8.4)
Not working due to permanent illness/ disability	41 (4.2)
Retired	186 (19.2)
Full-time homemaker/ looking after family	29 (3.0)
Student	34 (3.5)
Other/ don't know	8 (0.8)
**Annual combined net income for household**	
Under €19,999	115 (11.9)
€20,000 to €29,999	109 (11.2)
€30,000 to €49,999	227 (23.4)
€50,000 to €79,999	206 (21.3)
€80,000 or greater	128 (13.2)
Don’t know/ Refused to answer	184 (19.0)

There were similar proportions of men (48.1%) and women (51.7%). Participants ranged in age from 18–91 years, with a mean age of 47.9 (SD = 17.2). Over half of the sample had completed third-level education and worked as employees (either full-time or part-time).

### Healthcare seeking behaviour during COVID-19 restrictions

Of the 969 participants, 152 (15.7%) reported that they deliberately/consciously avoided contact with their G.P. about non COVID-19 related concerns while 81 (8.4%) reported that they avoided going to the hospital with a non COVID-19 related concern or health problem. The sociodemographic characteristics of the participants who avoided G.P. and hospital-based healthcare are outlined in Table 2.

**Table 2.** Socio-demographic characteristics of participants who avoided G.P. and hospital-based healthcare.

	Avoided G.P. (n=152) N (%)	Avoided hospital-based healthcare (n=81) N (%)
**Gender**		
Men ( *n* = 466)	51 (10.9)	28 (6.0)
Women ( *n* = 501)	100 (20.0)	53 (10.6)
**Age group**		
18–29 years ( *n* = 167)	23 (13.8)	7 (4.2)
30–39 years ( *n* = 167)	29 (17.4)	14 (8.4)
40–49 years ( *n* = 182)	37 (20.3)	16 (8.8)
50–59 years ( *n* = 157)	28 (17.8)	19 (12.1)
60–69 years ( *n* = 157)	20 (12.7)	7 (4.5)
70 years + ( *n* = 127)	13 (10.2)	17 (13.4)
**Highest level of education**		
Primary Level & Group/ Inter/ Junior Certificate ( *n* = 117)	12 (10.3)	16 (13.7)
Leaving Certificate ( *n* = 188)	27 (14.4)	19 (10.1)
Other Second Level/ PLC Cert or similar ( *n* = 92)	11 (12.0)	8 (8.7)
Third Level Degree/ Postgraduate Course ( *n* = 566)	101 (17.8)	38 (6.7)
**Employment status**		
Working as employee (full-time or part-time) ( *n* = 500)	79 (15.8)	37 (7.4)
Self-employed ( *n* = 90)	12 (13.3)	10 (11.1)
Unemployed/ Seeking work ( *n* = 81)	18 (22.2)	3 (3.7)
Not working due to permanent illness/ disability ( *n* = 41)	11 (26.8)	5 (12.2)
Retired ( *n* = 186)	21 (11.3)	17 (9.1)
Full-time homemaker/ looking after family ( *n* = 29)	6 (20.7)	5 (17.2)
Student ( *n* = 34)	4 (11.8)	4 (11.8)

One-fifth of all female participants ( *n* = 100; 20.0%) and those aged 40–49 years ( *n* = 37; 20.3%) reported avoiding contact with their G.P. More than a quarter of the participants who were not working due to illness/disability ( *n* = 11; 26.8%) also avoided contact with their G.P., although the overall number of participants in this grouping was small.

Just over 10% of all female participants ( *n* = 53) and 13% of those aged 70 years and above ( *n* = 17) reported avoiding going to hospital for non COVID-19 related concerns.

Table 3 shows data on self- reported health status, health related behaviours and exposures, perceived risk of COVID-19 and perceived susceptibility of contracting COVID-19 by self-reported health care avoidance status. Table 4 presents the findings from univariate and multivariate analyses on associations between these variables and avoidance of G.P. care. Similarly, Table 5 presents the findings from univariate and multivariate analyses on associations between these variables and avoidance of hospital care.

**Table 3.** Health status, health related behaviours and exposures and COVID-19 risk perceptions of participants who avoided G.P. and hospital-based healthcare.

	Avoided G.P. (n=152) N (%)	Avoided hospital-based healthcare (n=81) N (%)
**General health status**		
Excellent ( *n* = 163)	17 (10.4)	7 (4.3)
Very good ( *n* = 335)	48 (14.3)	18 (5.4)
Good ( *n* = 321)	48 (15.0)	28 (8.7)
Fair ( *n* = 118)	27 (22.9)	19 (16.1)
Poor ( *n* = 31)	12 (38.7)	9 (29.0)
**Alcohol consumption**		
Do not drink alcohol ( *n* = 227)	40 (17.6)	19 (8.4)
Occasional drinker ( *n* = 471)	63 (13.4)	43 (9.1)
Moderate drinker ( *n* = 251)	45 (17.9)	17 (6.8)
Heavy drinker ( *n* = 20)	4 (20.0)	2 (10.0)
**Smoking**		
Yes ( *n* = 151)	28 (18.5)	18 (11.9)
No ( *n* = 818)	124 (15.2)	63 (7.7)
**Adverse life event (<3 months)**		
Yes ( *n* = 287)	70 (24.4)	32 (11.1)
No ( *n* = 682)	82 (12.0)	49 (7.2)
**Increased risk of infection**		
Yes ( *n* = 56)	18 (32.1)	14 (25.0)
No ( *n* = 910)	134 (14.7)	67 (7.3)
**Perceived likelihood of contracting ** **coronavirus disease (COVID-19)**		
Strongly agree ( *n* = 123)	21 (17.1)	12 (9.8)
Tend to agree ( *n* = 292)	55 (18.8)	32 (11.0)
Tend to disagree ( *n* = 365)	52 (14.2)	27 (7.4)
Strongly disagree ( *n* = 165)	20 (12.1)	9 (5.5)
Don’t know ( *n* = 24)	4 (16.7)	1 (4.2)
**Perceived likelihood that COVID-19 would ** **be serious illness**		
Strongly agree ( *n* = 406)	69 (17.0)	48 (11.8)
Tend to agree ( *n* = 283)	52 (18.4)	19 (6.7)
Tend to disagree ( *n* = 199)	19 (9.5)	11 (5.5)
Strongly disagree ( *n* = 68)	10 (14.7)	3 (4.4)
Don’t know ( *n* = 13)	2 (15.4)	0 (0)

Female gender, self-reported health status and an adverse life event in the preceding 3-months were associated with avoidance of G.P. care in both univariate and multivariate analyses, whereas the association with increased risk of infection observed in univariate analyses was not observed in multivariate analyses, reflecting collinearity with self-reported health status, Table 4.

**Table 4.** Risk ratios (RR) for factors associated with self-reported avoidance of GP based healthcare in univariate and multivariate analyses.

	Univariate		Multivariate	
Variable	Risk ratio (confidence interval)	P value	Risk ratio (confidence interval)	P value
**Gender**				
Men	Reference			
Women	2.03 (1.41 – 2.93)	<0.001	1.77 (1.30 – 2.43)	<0.001
**Age group**				
18–29	Reference			
30–39	1.32 (0.73 – 2.39)	0.366	1.36 (0.83 – 2.23)	0.223
40–49	1.62 (0.92 – 2.86)	0.097	1.49 (0.91 – 2.43)	0.110
50–59	1.37 (0.75 – 2.50)	0.305	1.14 (0.67 – 1.93)	0.628
60–69	0.91 (0.48 – 1.74)	0.784	0.90 (0.50 – 1.61)	0.722
70–100	0.71 (0.35 – 1.47)	0.361	0.70 (0.36 - 1.35)	0.282
Trend	0.94 (0.84 – 1.04)	0.218	0.92 (0.84 – 1.01)	0.085
**Self-rated health status**				
Excellent	Reference			
Very good	1.45 (0.80 – 2.60)	0.219	1.35 (0.81 – 2.26)	0.250
Good	1.52 (0.84 – 2.73)	0.166	1.29 (0.76 – 2.21)	0.347
Fair	2.55 (1.32 – 4.93)	0.006	1.95 (1.05 – 3.61)	0.033
Poor	5.42 (2.25 – 13.08)	<0.001	2.83 (1.32 – 6.09)	0.008
Trend	1.39 (1.18 – 1.65)	<0.001	1.21 (1.02 – 1.43)	0.025
**Alcohol consumption**				
Do not drink	Reference			
Occasional drinker	0.72 (0.46 – 1.10)	0.130	0.68 (0.47 – 0.97)	0.035
Moderate drinker	1.01 (0.63 – 1.62)	0.966	1.13 (0.77 – 1.67)	0.525
Heavy drinker	1.16 (0.37 – 3.64)	0.804	0.79 (0.26 – 2.41)	0.678
**Smoking**				
No	Reference			
Yes	1.28 (0.81 – 2.01)	0.284	1.07 (0.74 – 1.56)	0.711
**Adverse life event (<3 months)**				
No	Reference			
Yes	2.38 (1.67 – 3.39)	<0.001	1.70 (1.27 – 2.28)	<0.001
**Increased risk of infection**				
No	Reference			
Yes	2.74 (1.52 – 4.95)	0.001	1.24 (0.75 – 2.08)	0.402
**COVID would be a serious illness for me**				
Strongly disagree	Reference			
Tend to disagree	0.61 (0.27 – 1.39)	0.241	0.44 (0.22 – 0.91)	0.027
Tend to agree	1.32 (0.63 – 2.75)	0.463	0.91 (0.47 – 1.75)	0.776
Strongly agree	1.19 (0.58 – 2.45)	0.634	0.86 (0.44 – 1.70)	0.672
Trend	1.19 (0.98 – 1.44)	0.078	1.14 (0.94 – 1.37)	0.186
**I am likely to catch COVID**				
Strongly disagree	Reference			
Tend to disagree	1.20 (0.69 – 2.09)	0.509	1.19 (0.72 – 1.90)	0.494
Tend to agree	1.69 (0.97 – 2.93)	0.063	1.56 (0.94 – 2.58)	0.085
Strongly agree	1.52 (0.78 – 2.96)	0.214	1.21 (0.65 – 2.23)	0.544
Trend	1.20 (0.99 – 1.45)	0.062	1.09 (0.93 – 1.28)	0.305

In univariate analyses, the following factors were associated with avoidance of hospital-based care: female gender, older age-group, poorer self-reported health status, an adverse life event in the preceding 3-months and those at an increased risk of infection, Table 5. In multivariate analyses, only individuals aged over 70 years were more likely to report avoidance of hospital-based care and the overall trend with age was non-significant. Poor self-rated health status was the only other factor that remained significant (trend *p* = 0.004), Table 5.

**Table 5.** Risk ratios (RR) for factors associated with self-reported avoidance of hospital-based healthcare in univariate and multivariate analyses.

	Crude		Adjusted	
Variable	Risk ratio (confidence interval)	P value	Risk ratio (confidence interval)	P value
**Gender**				
Men	Reference			
Women	1.85 (1.15 - 2.99)	0.011	1.50 (0.94 – 2.41)	0.091
**Age**				
18–29	Reference			
30–39	2.11 (0.83 – 5.36)	0.118	1.87 (0.81 – 4.31)	0.144
40–49	2.20 (0.88 – 5.50)	0.090	1.83 (0.80 – 4.20)	0.151
50–59	3.15 (1.28 – 7.71)	0.012	2.08 (0.94 – 4.60)	0.072
60–69	1.07 (0.37 – 3.11)	0.906	0.82 (0.30 – 2.23)	0.694
70–100	3.53 (1.42 – 8.80)	0.007	2.42 (1.08 – 5.44)	0.032
Trend	1.14 (0.99 – 1.31)	0.063	1.08 (0.95 – 1.23)	0.241
Health status *				
Excellent	Reference			
Very good	1.27 (0.52 - 3.10)	0.601	1.14 (0.48 – 2.60)	0.768
Good	2.13 (0.91 - 4.99)	0.082	1.69 (0.73 – 3.93)	0.220
Fair	4.28 (1.73 - 10.55)	0.002	2.42 (0.98 – 6.00)	0.056
Poor	9.12 (3.08 - 26.95)	<0.000	3.46 (1.30 – 9.10)	0.013
Trend	1.78 (1.42 – 2.26)	<0.001	1.38 (1.11 -1.71)	0.004
**Alcohol consumption**				
Do not drink	Reference			
Occasional drinker	1.10 (0.63 – 1.93)	0.741	1.18 (0.71 – 1.96)	0.512
Moderate drinker	0.80 (0.40 – 1.58)	0.517	0.98 (0.52 – 1.80)	0.951
Heavy drinker	1.22 (0.26 – 5.64)	0.802	1.11 (0.30 – 4.06)	0.873
**Smoking**				
No	Reference			
Yes	1.62 (0.93 – 2.82)	0.089	1.49 (0.88 – 2.55)	0.141
**Adverse life event (<3 months)**				
No	Reference			
Yes	1.62 (1.01 – 2.59)	0.044	1.28 (0.83 – 1.97)	0.269
**Increased risk of infection**				
No	Reference			
Yes	4.20 (2.19 – 8.09)	<0.001	1.57 (0.86 – 2.87)	0.140
**COVID would be a serious illness for me**				
Strongly disagree	Reference			
Tend to disagree	1.27 (0.34 – 4.69)	0.722	0.69 (0.19 – 2.45)	0.563
Tend to agree	1.57 (0.45 – 5.45)	0.481	0.74 (0.22 – 2.46)	0.624
Strongly agree	2.91 (0.88 – 9.61)	0.081	1.14 (0.35 – 3.75)	0.825
Trend	1.52 (1.16 – 2.01)	0.003	1.18 (0.87 – 1.61)	0.287
**I am likely to catch COVID**				
Strongly disagree	Reference			
Tend to disagree	1.39 (0.64 – 3.02)	0.408	1.87 (0.85 – 4.10)	0.118
Tend to agree	2.13 (0.99 – 4.59)	0.052	2.71 (1.20 – 6.10)	0.016
Strongly agree	1.87 (0.76 – 4.60)	0.170	1.81 (0.76 – 4.33)	0.182
Trend	1.28 (1.00 – 1.64)	0.052	1.21 (0.99 – 1.48)	0.061

## Discussion

This study of sociodemographic and health related factors associated with self-reported healthcare avoidance during the initial months of the COVID pandemic in Ireland has a number of significant findings with actionable lessons for the future. Women, those with self-reported poorer health status and those reporting the experience of an adverse life event in the previous three months, were at a higher risk of avoiding contact with their G.P. about non COVID-19 related concerns. Individuals at a higher risk of avoiding hospital-based care were those aged 70 years or older and who had poorer self-reported health status.

These findings are similar to those from a US study, in which women, older adults and those with a higher perceived risk of infection which likely reflects poorer self-assessed health status were more likely to avoid seeking healthcare ^7^.

The findings are also consistent with those from a cross-sectional study embedded within the ongoing population-based Rotterdam Cohort Study. In the latter study, women and individuals with lower perceived overall health and mental health issues (depression and anxiety) avoided seeking hospital-based healthcare, even when these groups had potentially serious symptoms (limb weakness, palpitations, and chest pain) ^14^. There is also evidence from a UK study of failure to seek medical attention during the pandemic among individuals with potential malignancy-related symptoms ^6^.

Thus the findings from this study complement and extend the available international literature on the impact of stay-at-home orders and related public health measures in response to a pandemic on healthcare utilisation avoidance in the population. In particular, the findings augment the available evidence that the adverse effects of public health restrictions are more pronounced in those at greatest need for healthcare.

For individuals to feel safe and able to access healthcare in future pandemics in Ireland, it is crucial that we note and learn the lessons from our experience with COVID-19. It seems clear that a significant proportion of vulnerable individuals weigh the risk of attending hospitals or GP surgery as higher than the risk of remaining at home with potentially serious issues. Post-pandemic studies have shown that these issues include late-stage, potentially curable, cancers ^8^. In Ireland, the National Cancer Registry reported a 10% shortfall in expected new diagnoses of cancer in 2020, improving to a 6% shortfall in 2021 ^15^. These issues may be especially relevant to patients with blood cancer, where the nature of the cancer, which affects the bone marrow and immune system, means that they are the most vulnerable group to opportunistic infections.

Our data provide important information specific to Ireland on behaviours during the pandemic. Because this study was carried out during periods of lockdown, recall bias was minimized and respondents answered regarding behaviours in real time. These valuable data provide the basis for targeted campaigns, specific information, and encouragement for vulnerable groups to help improve outcomes in future pandemics. The findings from this study suggest that people who may be most in need of health care are those who are least likely to access it.

This study had several limitations. The estimated response rate, although relatively high for a population-based telephone survey (43.6%), is clearly sub-optimal and the weighted sample of respondents may not be adequately representative of the underlying base population. In particular, it is likely that specific vulnerable sub-groups such as the homeless community are underrepresented in the study.

Lessons can be drawn for future pandemic preparedness in Ireland. Our data supports the use of advance planning to target vulnerable groups with appropriate public information campaigns on the appropriate response to potentially significant symptoms and concerns. Planning could also include work on the infrastructure required to pivot quickly to telephone clinics and video consultations, including work on data sharing issues, where appropriate.

## Data Availability

Zenodo: Factors influencing the likelihood of accessing healthcare during the COVID-19 pandemic in Ireland: lessons for the future. https://doi.org/10.5281/zenodo.10073077 ^12^ This project contains the following underlying data: National Household Survey – Wave 1, Ver. 2.sav Harvard Dataverse: Questionnaires for Surveys WP1 and WP2. https://doi.org/10.7910/DVN/EKUTFF ^11^ This project contains the following extended data: Survey 1 questionnaire in DOCX format (Appendix I) Survey 2 questionnaire in DOCX format (Appendix II) Data are available under the terms of the Creative Commons Attribution 4.0 International license (CC-BY 4.0).
