# Supplementary material for: Factors associated with self-reported healthcare utilization avoidance during the COVID-19 pandemic in Ireland: lessons for the future
Source: HRB Open Res. 2025 Dec 25;7:11. Originally published 2024 Feb 26. [Version 3] doi: 10.12688/hrbopenres.13829.3 (PMC12595297; doi:10.12688/hrbopenres.13829.3)
Supplement: Peer review history

# Reviewer report: Reviewer response for version 3

**DOI:** 10.21956/hrbopenres.15766.r52454

**Published:** 2025-12-30

**Authors:** Arkalgud Ramaprasad

**Affiliations:** 1 University of Illinois at Chicago, Chicago, IL, USA

The revisions are satisfactory. Thank you,

Is the work clearly and accurately presented and does it cite the current literature?

Yes

If applicable, is the statistical analysis and its interpretation appropriate?

Yes

Are all the source data underlying the results available to ensure full reproducibility?

Yes

Is the study design appropriate and is the work technically sound?

Yes

Are the conclusions drawn adequately supported by the results?

Partly

Are sufficient details of methods and analysis provided to allow replication by others?

Yes

Reviewer Expertise:

Healthcare, information systems, ontologies

I confirm that I have read this submission and believe that I have an appropriate level of expertise to confirm that it is of an acceptable scientific standard.

# Reviewer report: Reviewer response for version 2

**DOI:** 10.21956/hrbopenres.15553.r50942

**Published:** 2025-11-21

**Authors:** Arkalgud Ramaprasad

**Affiliations:** 1 University of Illinois at Chicago, Chicago, IL, USA

The authors have addressed one of my comments well but decided that addressing the second is beyond the scope of the paper.  I defer to the authors' judgment and accept the paper without reservation.

Is the work clearly and accurately presented and does it cite the current literature?

Yes

If applicable, is the statistical analysis and its interpretation appropriate?

Yes

Are all the source data underlying the results available to ensure full reproducibility?

Yes

Is the study design appropriate and is the work technically sound?

Yes

Are the conclusions drawn adequately supported by the results?

Partly

Are sufficient details of methods and analysis provided to allow replication by others?

Yes

Reviewer Expertise:

Healthcare, information systems, ontologies

I confirm that I have read this submission and believe that I have an appropriate level of expertise to confirm that it is of an acceptable scientific standard.

# Reviewer report: Reviewer response for version 2

**DOI:** 10.21956/hrbopenres.15553.r50944

**Published:** 2025-11-08

**Authors:** Anthony Kwame Morgan

**Affiliations:** 1 Kwame Nkrumah University of Science and Technology, Kumasi, Ashanti Region, Ghana

The revisions have been integrated.

 Thank you.

Is the work clearly and accurately presented and does it cite the current literature?

Yes

If applicable, is the statistical analysis and its interpretation appropriate?

Yes

Are all the source data underlying the results available to ensure full reproducibility?

Yes

Is the study design appropriate and is the work technically sound?

Yes

Are the conclusions drawn adequately supported by the results?

Yes

Are sufficient details of methods and analysis provided to allow replication by others?

Yes

Reviewer Expertise:

Health services research, ageing, geriatrics

I confirm that I have read this submission and believe that I have an appropriate level of expertise to confirm that it is of an acceptable scientific standard, however I have significant reservations, as outlined above.

# Reviewer report: Reviewer response for version 2

**DOI:** 10.21956/hrbopenres.15553.r50943

**Published:** 2025-11-08

**Authors:** Noel McCarthy

**Affiliations:** 1 Trinity College Dublin, Dublin, Ireland; 2 University of Warwick, Coventry, England, UK

Recommended changes on more explicit description of methods were not made. This is in the context of approving the work overall at the first stage.

Is the work clearly and accurately presented and does it cite the current literature?

Yes

If applicable, is the statistical analysis and its interpretation appropriate?

Partly

Are all the source data underlying the results available to ensure full reproducibility?

Yes

Is the study design appropriate and is the work technically sound?

Yes

Are the conclusions drawn adequately supported by the results?

Partly

Are sufficient details of methods and analysis provided to allow replication by others?

Yes

Reviewer Expertise:

Epidemiology,

I confirm that I have read this submission and believe that I have an appropriate level of expertise to confirm that it is of an acceptable scientific standard.

# Reviewer report: Reviewer response for version 1

**DOI:** 10.21956/hrbopenres.15148.r39734

**Published:** 2024-05-31

**Authors:** Arkalgud Ramaprasad

**Affiliations:** 1 University of Illinois at Chicago, Chicago, IL, USA

The title of the paper is: “Factors influencing the likelihood of accessing healthcare during the COVID-19 pandemic in Ireland: lessons for the future.” The strength of the paper is its description of the factors influencing the object of the study; the weakness is that it does not explore or explain how these factors exert their influence. How do these factors result in drivers of access, set norms for access, or barriers to access? Thus, while the descriptive value of the research is high, its explanatory value is low. Consequently, its predictive value for managing access in the future, intervention value for improving access in the future through feedback is low. Thus, the paper as is it now is more about factors describing … and not factors influencing….

 The data the authors have will not allow them to analyze the influencing factors. However, could they do the following: (a) integrate the factors they have discovered into a coherent conceptual model, and (b) infer the potential barriers, norms, and drivers to access based on the model.

 The following are illustrative of the questions that may be insightful:

- Gender
  - Why do a greater percentage of women avoid both GP visit (20%) and hospital-based healthcare (10.6%) than men (10.9% GP Visit, 6.0% Hospital).
  - What are the gender-based drivers of, norms for, barriers to men/women visiting the GP/Hospital?
- Highest level of education
  - Why do more educated people avoid GP visits (17.8%) and less educated people avoid hospital care (13.7%).
  - What are the education-based drivers of, norms for, and barriers to visiting the GP/Hospital?

Similar questions may be posed with reference to the other parameters in Tables 2-5. A coherent framework and answers to such questions derived from the study could help fulfill the promise of the title, at least partly.

Is the work clearly and accurately presented and does it cite the current literature?

Yes

If applicable, is the statistical analysis and its interpretation appropriate?

Yes

Are all the source data underlying the results available to ensure full reproducibility?

Yes

Is the study design appropriate and is the work technically sound?

Yes

Are the conclusions drawn adequately supported by the results?

Partly

Are sufficient details of methods and analysis provided to allow replication by others?

Yes

Reviewer Expertise:

Healthcare, information systems, ontologies

I confirm that I have read this submission and believe that I have an appropriate level of expertise to confirm that it is of an acceptable scientific standard, however I have significant reservations, as outlined above.

# Response

**Published:** 2025-10-24

**Authors:** Mary Joyce

**Affiliations:** National Suicide Research Foundation, Ireland

1. The title of the paper is: “Factors influencing the likelihood of accessing healthcare during the COVID-19 pandemic in Ireland: lessons for the future.” The strength of the paper is its description of the factors influencing the object of the study; the weakness is that it does not explore or explain how these factors exert their influence. How do these factors result in drivers of access, set norms for access, or barriers to access? Thus, while the descriptive value of the research is high, its explanatory value is low. Consequently, its predictive value for managing access in the future, intervention value for improving access in the future through feedback is low. Thus, the paper as is it now is more about factors describing … and not factors influencing….

**Response: **We thank the reviewer for these insightful comments and we fully accept the thrust of his argument that the paper is primarily descriptive with limited explanatory value. In the title of the revised manuscript we have substituted *factors influencing.. *with *factors associated with…., *as detailed in the response to reviewer #2. 

1. The data the authors have will not allow them to analyze the influencing factors. However, could they do the following: (a) integrate the factors they have discovered into a coherent conceptual model, and (b) infer the potential barriers, norms, and drivers to access based on the model.The following are illustrative of the questions that may be insightful:

- Gender
  - Why do a greater percentage of women avoid both GP visit (20%) and hospital-based healthcare (10.6%) than men (10.9% GP Visit, 6.0% Hospital).
  - What are the gender-based drivers of, norms for, barriers to men/women visiting the GP/Hospital?
- Highest level of education
  - Why do more educated people avoid GP visits (17.8%) and less educated people avoid hospital care (13.7%).
  - What are the education-based drivers of, norms for, and barriers to visiting the GP/Hospital?

1. Similar questions may be posed with reference to the other parameters in Tables 2-5. A coherent framework and answers to such questions derived from the study could help fulfill the promise of the title, at least partly.

**Response: **Response to points 2 & 3: While we agree that the suggested conceptual models would be interesting and useful, we believe that they are beyond the scope of the current paper.

# Reviewer report: Reviewer response for version 1

**DOI:** 10.21956/hrbopenres.15148.r39062

**Published:** 2024-05-09

**Authors:** Anthony Kwame Morgan

**Affiliations:** 1 Kwame Nkrumah University of Science and Technology, Kumasi, Ashanti Region, Ghana

I have carefully reviewed your manuscript, and I would like to offer some constructive feedback for improvement.

1. Alignment of Topic and Objective: There seems to be a disconnection between the topic and the objective of your study. It is unclear whether you examined healthcare utilization avoidance or healthcare utilization during COVID-19. I recommend clarifying this to ensure coherence between the topic and objective.
2. Discussion Content: The discussion section lacks depth and fails to provide a thorough analysis of the results. Merely citing other studies without providing an explanation of why the results occurred is insufficient. I suggest expanding the discussion to include plausible reasons for the observed outcomes, drawing on relevant health behavior theories to strengthen the implications of your findings.
3. Grammar and Construction: There are several grammatical and construction errors throughout the manuscript that need to be addressed for clarity and readability. I recommend thorough proofreading and revision to improve the overall quality of the writing.

I believe that addressing these issues will significantly enhance the clarity and impact of your manuscript. I look forward to seeing the revised version.

Is the work clearly and accurately presented and does it cite the current literature?

Yes

If applicable, is the statistical analysis and its interpretation appropriate?

Yes

Are all the source data underlying the results available to ensure full reproducibility?

Yes

Is the study design appropriate and is the work technically sound?

Yes

Are the conclusions drawn adequately supported by the results?

Yes

Are sufficient details of methods and analysis provided to allow replication by others?

Yes

Reviewer Expertise:

Health services research, ageing, geriatrics

I confirm that I have read this submission and believe that I have an appropriate level of expertise to state that I do not consider it to be of an acceptable scientific standard, for reasons outlined above.

# Response

**Published:** 2025-10-24

**Authors:** Mary Joyce

**Affiliations:** National Suicide Research Foundation, Ireland

I have carefully reviewed your manuscript, and I would like to offer some constructive feedback for improvement.

1. Alignment of Topic and Objective: There seems to be a disconnection between the topic and the objective of your study. It is unclear whether you examined healthcare utilization avoidance or healthcare utilization during COVID-19. I recommend clarifying this to ensure coherence between the topic and objective.

**Response: **We thank the reviewer for this observation. The focus of the study was on healthcare utilisation avoidance, based on responses to the following two questions:

 During the period of restricted movement / lockdown, have you done any of the following?

 - “Deliberately / consciously avoided contacting your GP (General Practitioner) about non-coronavirus concerns or problems that you would normally bring to his/her attention.”

 - “Deliberately/consciously avoided going to the hospital with a non-coronavirus concern or health problem that would in normal circumstances require a visit to the hospital.”

 In the revised manuscript we have changed the title from “ *Factors influencing the likelihood of accessing healthcare during the COVID-19 pandemic in Ireland: lessons for the future*” to “ ***Factors associated with self-reported healthcare utilization avoidance during the COVID-19 pandemic in Ireland: lessons for the future”.***

1. Discussion Content: The discussion section lacks depth and fails to provide a thorough analysis of the results. Merely citing other studies without providing an explanation of why the results occurred is insufficient. I suggest expanding the discussion to include plausible reasons for the observed outcomes, drawing on relevant health behavior theories to strengthen the implications of your findings.

**Response: **The primary focus of this paper is on the magnitude and distribution of the effects of pandemic restrictions on health care utilisation avoidance. In the current manuscript, we refer briefly in the discussion to the likely impact on behaviour of differential weighting of the risk of staying at home versus presenting for primary or secondary care and the associated need for relevant public information campaigns. We believe that further speculation on psychological determinants of health care avoidance and the relevant behaviour change implications of the findings is beyond the scope of this paper.

1. Grammar and Construction: There are several grammatical and construction errors throughout the manuscript that need to be addressed for clarity and readability. I recommend thorough proofreading and revision to improve the overall quality of the writing.

I believe that addressing these issues will significantly enhance the clarity and impact of your manuscript. I look forward to seeing the revised version.

**Response:** We have carefully reviewed and revised the manuscript in the light of these comments.

# Reviewer report: Reviewer response for version 1

**DOI:** 10.21956/hrbopenres.15148.r39055

**Published:** 2024-05-09

**Authors:** Noel McCarthy

**Affiliations:** 1 Trinity College Dublin, Dublin, Ireland; 2 University of Warwick, Coventry, England, UK

This is a good study generally clearly reported and with data and instruments in the public domain. The results are useful, my suggestions for edits are relatively minor as given below.

 The study design is described as “A nationally representative cross-sectional telephone survey was conducted …” while in the analysis it states that - “the estimates were weighted to account for the survey sampling design”. These seem to contradict each other. Looking at the cited methodology paper by Troya et al I again cannot find any methodological reference to weights. I think that this could be made clearer.

 Self-rated health is dropped from the model due to reported collinearity with self-perceived risk of infection. No processes for this, or generally for model building, are given in the methods. On univariate analysis both had strong and statistically significant associations with healthcare avoidance including a dose response association for self-rated health. At a guess - if the other decision had been made the authors would be reporting that self-rated health strongly and perhaps in a dose response fashion predicted healthcare avoidance. Given that this effectively steers what is concluded and communicated so substantially it would be good to describe the process leading to the decision on which to drop.

 The authors emphasise in the results text and even note in the abstract that “participants who agreed that they were likely to catch COVID-19 were at a higher risk of avoiding going to hospital than those who strongly disagreed (RR: 2.80 (1.25 – 6.25)”. However, this was the result for “tend to agree” while those who “strongly agree” had a non-significant and substantially weaker (RR 1.91) association. This feels like cherry picking. If the process had included an overall assessment for association with the variable before drilling into the specific association in this band it might be more convincing. Somewhat similarly for age where e.g. the significant, but marginally so in the context of extensive testing, association between being 50-59 years old and avoiding healthcare is picked out and even mentioned in the abstract. Here again an overall test of whether the age categories used showed association with outcome before going into individual band would seem a more solid statistical approach.

 In the discussion the highlighting of these findings as “especially relevant to patients with blood cancer” appears arbitrary to me for a study that related to self-perceived risk. Additionally, agreeing with the authors claim in general that their results give “actionable lessons for the future” this perhaps applies least so such a patient group which has such direct supervision and intense support evidenced by the anecdotal evidence for such that the authors note in the discussion.

Is the work clearly and accurately presented and does it cite the current literature?

Yes

If applicable, is the statistical analysis and its interpretation appropriate?

Partly

Are all the source data underlying the results available to ensure full reproducibility?

Yes

Is the study design appropriate and is the work technically sound?

Yes

Are the conclusions drawn adequately supported by the results?

Partly

Are sufficient details of methods and analysis provided to allow replication by others?

Yes

Reviewer Expertise:

Epidemiology,

I confirm that I have read this submission and believe that I have an appropriate level of expertise to confirm that it is of an acceptable scientific standard, however I have significant reservations, as outlined above.

# Response

**Published:** 2025-10-24

**Authors:** Mary Joyce

**Affiliations:** National Suicide Research Foundation, Ireland

We thank the three reviewers for their positive and constructive engagement with our work and for their commentary which has considerably enhanced the paper. We provide our responses to the reviewers comments below and outline how these have been addressed in the revised paper.

**Response to Noel McCarthy**

 This is a good study generally clearly reported and with data and instruments in the public domain. The results are useful, my suggestions for edits are relatively minor as given below.

1. The study design is described as “A nationally representative cross-sectional telephone survey was conducted …” while in the analysis it states that - “the estimates were weighted to account for the survey sampling design”. These seem to contradict each other. Looking at the cited methodology paper by Troya et al I again cannot find any methodological reference to weights. I think that this could be made clearer.

**Response: **We apologise for the lack of information and confusion created about the weights. This information was originally outlined in Troya et al. (2022) but was inadvertently omitted from this manuscript. Data were weighted by age, gender, and region, with population estimates based on the Irish Labour Force Survey. We applied the same weights for this analysis. The manuscript has been updated to include this information. We now cite both the Troya 2022 paper and the Irish Labour Force Survey in the Methods section.

1. Self-rated health is dropped from the model due to reported collinearity with self-perceived risk of infection. No processes for this, or generally for model building, are given in the methods. On univariate analysis both had strong and statistically significant associations with healthcare avoidance including a dose response association for self-rated health. At a guess - if the other decision had been made the authors would be reporting that self-rated health strongly and perhaps in a dose response fashion predicted healthcare avoidance. Given that this effectively steers what is concluded and communicated so substantially it would be good to describe the process leading to the decision on which to drop.

**Response: **We thank the reviewer for this observation. In the first instance we should clarify that the “risk of infection” variable is based on objective criteria, specifically cocooning because of diabetes, cancer, a severe respiratory condition, a condition with a very high risk of infections, or being on medication that increased the likelihood of contracting infections. The definition of this variable is now clearly described in the Measures section of the Method.

 As one would anticipate, this variable is highly correlated with self-rated health status, with those characterised as at increased risk of infection more likely to rate their health as ‘fair’ or ‘poor’ when compared with those not at increased risk of infection, see table below:

**Increased risk of infection**

**Health Status** *No* (n = 913) *Yes *(n = 56) *Total *(n = 968)

 Excellent               163 (18%)           0 (0%)         163 (17%)

 Very Good             325 (36%)        10 (18%)        335 (35%)

 Good                     307 (34%)        14 (25%)        321 (33%)

 Fair                        100 (11%)        18 (32%)        118 (12%)

 Poor                          18 (2%)        13 (23%)         31 (3%)

 Each of these variables (self-rated health status and increased risk of infection) was associated with GP and hospital avoidance in multivariate analyses when the other variable was excluded. However, in multivariate analyses with inclusion of both variables, only self-rated health status was associated with GP and hospital avoidance. With the benefit of hindsight, we made assumptions about multicollinearity in the data that were not adequately tested, and it is now clear that self-rated health is an important predictor of GP and hospital avoidance in these data. The relevant text in the manuscript, including the abstract, has been revised accordingly.

1. The authors emphasise in the results text and even note in the abstract that “participants who agreed that they were likely to catch COVID-19 were at a higher risk of avoiding going to hospital than those who strongly disagreed (RR: 2.80 (1.25 – 6.25)”. However, this was the result for “tend to agree” while those who “strongly agree” had a non-significant and substantially weaker (RR 1.91) association. This feels like cherry picking. If the process had included an overall assessment for association with the variable before drilling into the specific association in this band it might be more convincing. Somewhat similarly for age where e.g. the significant, but marginally so in the context of extensive testing, association between being 50-59 years old and avoiding healthcare is picked out and even mentioned in the abstract. Here again an overall test of whether the age categories used showed association with outcome before going into individual band would seem a more solid statistical approach.

**Response: **We agree with the reviewer that we should have tested for and reported on the overall association with the COVID-19 susceptibility variable and age in these analyses. In further analyses which included tests for linear trend, age and COVID-19 susceptibility was non-significant for G.P. avoidance and hospital avoidance. The relevant text in the manuscript, including the abstract, has been revised accordingly.

1. In the discussion the highlighting of these findings as “especially relevant to patients with blood cancer” appears arbitrary to me for a study that related to self-perceived risk. Additionally, agreeing with the authors claim in general that their results give “actionable lessons for the future” this perhaps applies least so such a patient group which has such direct supervision and intense support evidenced by the anecdotal evidence for such that the authors note in the discussion.

**Response: **We understand that the reference to patients with blood cancer in the discussion seems arbitrary and that the “actionable lessons for the future” that arise from these data apply to all patient groups. However, in the overall context of cancer care during a pandemic, we believe that the unique vulnerability of patients with blood cancers arising from the bone marrow and immune system merits highlighting. This specific issue is not generally understood and appreciated. However, in the revised manuscript, the relevant section has been shortened considerably.
